# Internal carotid artery occlusion mimicking artery of Heubner stroke

**DOI:** 10.11604/pamj.2022.42.43.34826

**Published:** 2022-05-16

**Authors:** Jamir Pitton Rissardo, Ana Letícia Fornari Caprara

**Affiliations:** 1 Medicine Department, Federal University of Santa Maria, Santa Maria, Brazil

**Keywords:** Internal carotid artery, artery of Heubner, stroke

## Image in medicine

A 65-year-old male presenting with left upper and lower limbs weakness with twenty-four hours of onset was admitted to our hospital. The subject reported that the symptoms began after he rose quickly from a chair. He was a previously healthy farmer and his family history was negative for neurological diseases. The physical examination showed left hyperreflexia and plantar extension. Laboratorial tests were within normal limits. A cranial non-contrast CT scan was suggestive of infarct in the recurrent artery of Heubner (Axial (A)) view showing hypodense round area in right medial lenticulo-striate artery territory). Twenty-four hours after admission, the subject was apparently normal. Upon further questioning, he admitted that he had several similar episodes of weakness in the past year, which he thought were normal due to his age and daily work in agriculture. He only sought medical assistance this time because his daughter insisted. A cranial CT angiography revealed the complete occlusion of the right extracranial internal carotid artery (Axial (B), sagittal (C), and volume-rendered (D) views). This report supports the hypothesis that the internal watershed area is more affected by cerebral hypoperfusion than the area between the middle cerebral artery and anterior cerebral artery. Furthermore, it is proposed that any patient with a suggestive infarction of artery of Heubner should be thoroughly inquired about the recurrence of symptoms and the existence of triggers, since radiologic exams provide a presumptive diagnosis that should be carefully analyzed together with clinical manifestations.

**Figure 1:**
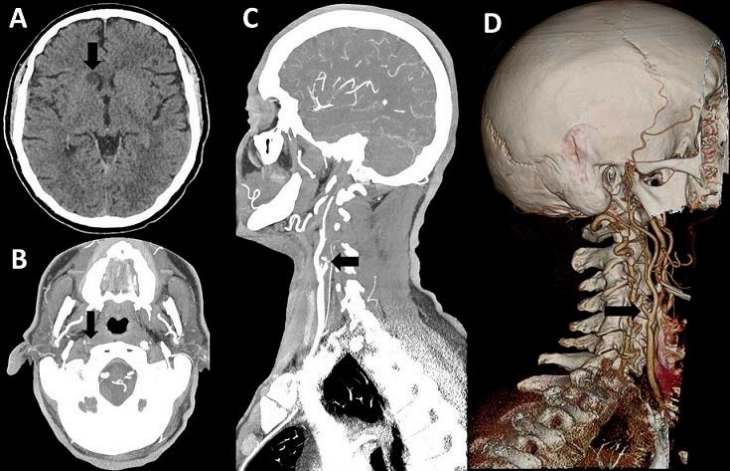
axial (A) view of cranial non-contrast CT scan showing hypodense round area in right medial lenticulo-striate artery territory, axial (B), sagittal (C), and volume-rendered (D) views of cranial CT angiography showing complete occlusion of right extracranial internal carotid artery

